# User evaluations offer promise for pod-intravaginal ring as a drug delivery platform: A mixed methods study of acceptability and use experiences

**DOI:** 10.1371/journal.pone.0197269

**Published:** 2018-05-14

**Authors:** Kate M. Guthrie, Rochelle K. Rosen, Sara E. Vargas, Melissa L. Getz, Lauren Dawson, Melissa Guillen, Jaime J. Ramirez, Marc M. Baum, Kathleen L. Vincent

**Affiliations:** 1 The Centers for Behavioral & Preventive Medicine, The Miriam Hospital, Providence, Rhode Island, United States of America; 2 Department of Psychiatry & Human Behavior, Alpert Medical School of Brown University, Providence, Rhode Island, United States of America; 3 Department of Behavioral & Social Sciences, Brown University School of Public Health, Providence, Rhode Island, United States of America; 4 Department of Obstetrics and Gynecology, University of Texas Medical Branch, Galveston, Texas, United States of America; 5 Department of Chemistry, Oak Crest Institute of Science, Monrovia, California, United States of America; Elizabeth Glaser Pediatric AIDS Foundation, UNITED STATES

## Abstract

**Background:**

Effective HIV prevention requires efficient delivery of safe and efficacious drugs and optimization of user adherence. The user’s experiences with the drug, delivery system, and use parameters are critical to product acceptability and adherence. Prevention product developers have the opportunity to directly control a drug delivery system and its impact on acceptability and adherence, as well as product efficacy. Involvement of potential users during preclinical design and development can facilitate this process. We embedded a mixed methods user evaluation study into a safety and pharmacokinetics (PK) trial of a pod-intravaginal ring delivering antiretroviral agents.

**Methodology:**

Women enrolled in two cohorts, ultimately evaluating the safety/PK of a pod-IVRs delivering TDF-alone, TDF-FTC, and/or TDF-FTC-MVC. A 7-day use period was targeted for each pod-IVR, regardless of drug or drug combination. During the clinical study, participants provided both quantitative (i.e., survey) and qualitative (i.e., in-depth interview) data capturing acceptability, perceptibility, and adherence behaviors. Initial sexual and reproductive health history surveys, daily diaries, a final acceptability and willingness to use survey, and a qualitative in-depth interview comprised the user evaluation data for each pod-IVR experienced by the participants.

**Findings:**

Overall, the majority of participants (N = 10) reported being willing to use the pod-IVR platform for HIV prevention should it advance to market. Confidence to use the pod-IVR (e.g., insertion, removal) was high. There were no differences noted in the user experience of the pod-IVR platform; that is, whether the ring delivered TDF-alone, TDF-FTC, or TDF-FTC-MVC, users’ experiences of the ring were similar and acceptable. Participants did report specific experiences, both sensory and behavioral, that impacted their use behaviors with respect to the ring, and which could ultimately impact acceptability and adherence. These experiences, and user evaluations elicited by them, could both challenge use or be used to leverage use in future trials and product rollout once fully articulated.

**Conclusions:**

High willingness-to-use data and lack of salient differences in user experiences related to use of the pod-IVR platform (regardless of agents delivered) suggests that the pod-IVR is a feasible and acceptable drug delivery device in and of itself. This finding holds promise both for an anti-HIV pod-IVR and, potentially, a multipurpose prevention pod-IVR that could deliver both prevention for sexually transmitted infections (STIs) including HIV and contraception. Given the very early clinical trial context, further acceptability, perceptibility, and adherence data should continue to be explored, in the context of longer use periods (e.g., 28-day ring use), and in the contexts of sexual activity and menses. Using early design and development contexts to gain insights into potential challenges and facilitators of drug delivery systems such as the pod-IVR could save valuable resources and time as a potential biomedical technology moves through the clinical trial pipeline and into real-world use.

## Introduction

Women continue to be greatly affected by the human immunodeficiency virus (HIV) pandemic, accounting for over half of all adults living with HIV worldwide.[1, 2] Women in the United States make up approximately 20% of new HIV diagnoses annually, with the majority (86%) attributing their HIV infection to heterosexual sex.[3] The current HIV prevention toolbox is limited to behavioral strategies (e.g. condom-protected sex; HIV testing; limiting number(s) of partners) and oral antiretroviral pre-exposure prophylaxis (PrEP). PrEP has been shown to be effective and safe in some populations. However, adherence has been a significant concern, both for PrEP and for topical vaginal and rectal HIV prevention technologies.[4–6]

HIV prevention product developers have worked over the past two decades to develop efficacious and effective HIV prevention methods that women can use with minimal or no need to negotiate that use with male sexual partners. While the majority of topical vaginal microbicide trials have been unsuccessful, the RING and ASPIRE trials evaluated a monthly intravaginal ring (IVR) containing dapivirine that showed significant decreases in HIV incidence, demonstrating partial efficacy of a sustained-release approach to delivering antiretroviral prophylaxis. The dapivirine ring significantly reduced incident HIV infections by more than half among women over age 21 years.[4, 6]

In addition to HIV-only prevention, there is a need for products that women can use for overall sexual and reproductive health needs. With this in mind, developers are working toward multipurpose prevention technologies (MPTs), single or multidrug formulations or devices targeting combinations of contraception, HIV prevention, and /or prevention of various STIs.[7] IVRs are marketed for contraception and hormone replacement therapy, and provide a unique and promising drug delivery system for MPTs. Depending on the structure and design of the IVR, they can deliver multiple agents at sustained, safe, yet potent concentrations over long durations. Thus, IVRs could offer a female-controlled method of prevention, either for HIV alone or as an MPT.

Effective prevention requires not only efficient delivery of safe and efficacious drugs, but also the optimization of user adherence. The user’s experience with the drug and its use parameters are critical to product acceptability and adherence. Current conceptualizations of adherence and acceptability do not fully articulate or account for patterns of use and non-use. Prevention product developers have the opportunity to directly control a drug delivery system’s impact on acceptability and adherence, as well as product efficacy. Indeed, prevention products can and should be developed so that they achieve performance standards for both behavioral and biological functions.[8–11]

The goal is to develop prevention products that empower women to protect themselves from HIV. Most believe that sustained-release IVR-delivered ART can be achieved. The purpose of the current study, set within the context of a pre-phase 1 safety and pharmacokinetic study of an innovative pod-IVR drug delivery system (described elsewhere)[12], was to begin to understand women’s experiences using a pod-IVR.[13] By incorporating user experience studies into the early product development process, developers will be able to identify any user-related concerns or difficulties with use, and thus have the opportunity to integrate effective use instructions and psychoeducation into clinical trials and subsequent marketing strategies. Developing prevention products that can optimize the user experience increases the likelihood that these products will be used consistently and correctly. With high levels of adherence, the impact on global public health has the potential to be far-reaching, decreasing HIV and STI incidence and prevalence.

## Methods

### Study design

The clinical trial which served as the context for this user evaluation study was approved by the University of Texas Medical Branch at Galveston IRB. This single-center nonrandomized, non-blinded Pre-Phase 1 study was conducted at the University of Texas Medical Branch (UTMB) from June 2015 to July 2016. Two cohorts of women completed 7-day pod-IVR safety and pharmacokinetics studies. The first cohort completed a 7-day use cycle with each of two pod-IVRs, the first containing tenofovir disoproxil fumarate alone (TDF-only pod-IVR), the second IVR containing tenofovir disoproxil fumarate (TDF) and emtricitabine (FTC) (TDF-FTC pod-IVR). This second TDF-FTC pod-IVR corresponds to the oral formulation (Truvada®), which is the only FDA-approved regimen for HIV PrEP. The second cohort completed a single 7-day use cycle, using a triple-drug combination of TDF, FTC, and maraviroc (MVC; TDF-FTC-MVC pod-IVR)). Details can be found at https://clinicaltrials.gov/ct2/show/NCT02431273.

### Study population / recruitment

#### Cohort 1 (June 2015-December 2015)

Healthy women between the ages of 18 and 45 were recruited to use two pod-IVRs, each for 7 days. Recruitment was conducted through flyers and electronic announcements and continued until six women were enrolled. Interested volunteers were screened over the phone by research staff to determine basic eligibility. If eligible after the phone screen, women attended an in-person clinical screening visit. Women were excluded from the study if they: self-reported or tested positive for HIV, Hepatitis B, genital herpes, and/or any vaginal infections; were pregnant or less than 6 months post-partum; and/or had a history of adverse reactions to any of the study drugs or materials. Study participants agreed to abstain from vaginal, anal, and oral sex during ring use and up to two days after ring removal; they also agreed not to use any vaginal products except for the study intravaginal ring (IVR) until after ring removal. Participants agreed to blood draws and vaginal exams throughout the course of the study. Details of the clinical aspects of study can be found elsewhere.[12]

#### Cohort 2 (March 2016-July 2016)

Following completion of the first cohort, women were enrolled in Cohort 2 (TDF-FTC-MVC pod-IVR). All inclusion/exclusion criteria were the same, with the addition of a history of adverse reactions to MVC, specifically. All procedures were parallel to those for Cohort 1, with the exception of women in Cohort 2 only having to complete a single 7-day pod-IVR use period.

### Study materials and procedures

The pod-IVR is an innovative drug delivery system.[13] Each pod-IVR is made primarily of silicone with drug pods embedded in the unmedicated silicone ring scaffold.[14, 15] The outside diameter of the pod-IVR measures 56 mm while the inside diameter is 40 mm. The cross-sectional diameter is 8.0 mm. The number of pods varies as a function of the drugs delivered, with as many as ten pods: empty pod cavities are back-filled with silicone. Drugs are released into the vaginal tissue from the drug pods via delivery channels that can be calibrated to specific dosing requirements. As noted above, three pod-IVR regimens were studied: a single drug (TDF-only) pod-IVR, a dual-drug (TDF-FTC) pod-IVR, and a triple-drug (TDF-FTC-MVC) pod-IVR. Women were asked to use each pod-IVR for which they were enrolled for 7 continuous days.

Prior to using any pod-IVR, each woman completed a sexual and reproductive health history survey. This survey captured psychosocial and behavioral data, including basic demographics, vaginal product use history, contraceptive use history, sexual relationship and behavior data, pregnancy history and HIV/STI testing history. With the start of each pod-IVR use period, the participants were given verbal instructions by the study clinician on how to insert the pod-IVR into their vagina; the study clinician confirmed proper placement with a digital exam following insertion. During the 7-day use period, women completed daily diaries and recorded any notable pod-IVR experiences, including physical symptoms/sensations or ring expulsions. Following the 7-day use period, participants completed a final survey capturing data related to their experiences with the pod-IVR, including the sensations and perceptions they noted throughout use. Surveys included conventional acceptability and behavior items, largely as frequencies and Likert scales. All Likert response formats were on 5-point scales. Agreement Likert benchmarks were: do not agree at all; agree a little; agree somewhat; agree a lot; agree completely. The Willingness-to-Use benchmarks were: definitely not; probably not; unsure; probably; definitely. Confidence Likert benchmarks were: not at all confident, not very confident, somewhat confident, very confident, completely confident. Importance Likert benchmarks were: not at all important, a little important, somewhat important, very important, and extremely important. The final survey was followed by a qualitative in-depth interview facilitated by trained research staff. The interview explored a participant’s experiences and opinions of the IVR and its use, including insertion, day-to-day use, removal, and thoughts regarding future use of sustained use pod-IVRs, and, in particular, asked women to consider their anticipations of use during sexual intercourse and menstruation.

All clinical procedures are described elsewhere.[12]

### Data analysis

All quantitative behavioral data were collected using a computer-assisted self-interview (CASI) format. Descriptive statistics were calculated in SPSS v.20. In-depth interviews were audio recorded and transcribed for applied thematic analysis[16] using a framework matrix.[17–19] Transcribed interviews were de-identified and reviewed for accuracy and then entered into NVivo 10 qualitative data analysis software,[20] which contains a framework matrix tool. A framework matrix is a case- and theme-based data reduction technique in which the qualitative analyst reviews each interview transcript and summarizes participants’ comments on key research themes into the matrix. Rows in the matrix represent each participant. Columns represent the major research themes.

The matrix approach enables both topical- and case-based thematic analysis. Each column provides access to all the participants’ experiences with respect to a particular topic (i.e., insertion of the first ring); each row summarizes one participant’s full experience in the study (i.e., all her experiences with each of the rings she used). Combining topics and cases enables rich understanding of the interface of the participants’ rings experiences, behavior and personal meaning making.[7] The framework matrix tool in NVivo also allows quick linkage to the original transcript data and provides access to participants’ own words when necessary.

In the current analysis, we present data which, for the first time, characterizes the user experience of a pod-IVR. We pay particular attention to those themes that illustrate the interaction between the pod-IVR as a drug delivery system and the user. Our goal was to characterize that experience and explore any differences in the user experience across the three rings.

## Results

### Participants

Across cohorts, 10 women participated. Table 1 presents de-identified listings of participants’ status within each pod-IVR drug condition (i.e., by cohort). Four women experienced pod-IVRs with TDF-alone and TDF-FTC; two women experienced pod-IVRs with TDF-alone, TDF-FTC, and TDF-FTC-MVC; and four women experienced the TDF-FTC-MVC pod-IVR only. The mean age was 27.8 years (range 21–41). Six reported being White/Caucasian, while 3 were Black/African-American and 1 did not respond to the race question. Three endorsed being Hispanic/Latina. Nine participants reported being “single, never married,” while 1 was divorced. With respect to highest educational attainment, 1 had a high school diploma, 3 had associate’s degrees, 5 had bachelor’s degrees, and 1 reported a graduate/professional degree. Two participants reported annual incomes below $15,000, while 5 reported annual incomes $15,000–36,000, and 3 reported annual incomes over $36,000.

**Table 1:** Participants by cohort and pod-IVR evaluated.

Cohort 1		Cohort 2
TDF pod-IVR	TDF-FTC pod-IVR	TDF-FTC-MVC pod-IVR
✔	✔	---
✔	✔	---
✔	✔	---
✔	✔	---
---	---	✔
---	---	✔
---	---	✔
---	---	✔

Each row represents a de-identified participant’s Cohort completions.

BMI (body mass index) averaged 30.8 (range 24–44). Eight reported being typical tampon users, while none (0) reported being typical menstrual cup users. Two women, one from each cohort, were parous; 8 were nulliparous. All participants were required to be using effective contraception: 8 were using oral contraceptive agents, 1 was using a hormonal IUD and 1 had been surgically sterilized. Five of the participants reported that they did not use condoms during vaginal sex, while 4 reported irregular use of condoms, and 1 reported using condoms “all the time.” Four reported a previous history of having been diagnosed with a sexually transmitted infection, and 5 reported being tested for HIV in the past year. Four reported some perception of STI or HIV risk, while the remainder reported no risk for sexually transmitted infections.

### Quantitative results

#### Willingness-to-use the pod-IVR

In willingness-to-use data (a proxy for acceptability), 6 of 10 women would probably/definitely use the pod-IVR if it were available for HIV prevention. Three of the four women that were “unsure” noted that they did not need HIV prevention. The fourth noted that her experience of cramping was uncommon for her and left her with concerns. All 10 women would probably/definitely recommend the pod-IVR to a friend. Five of the women in Cohort 1 reported that they would probably/definitely would use it if it was a 7- or 28-day ring: the remaining participant was “unsure.” All the women in Cohort 2, including the woman who was unsure in Cohort 1, would probably/definitely use the pod-IVR if it was a 7-day ring, and 5 of the 6 women would probably/definitely use it if it was a 28-day ring (the woman who was unsure regarding 28-day pod-IVR use in Cohort 1was among those five). Interestingly, responses to willingness-to-use the ring began to spread across the scale responses as anticipations of use time increased: thus, fewer women reported being as willing to use 3- and 6-month rings; some responded that they would not use a 3- or 6-month IVR.

When asked (via open text questions) what they liked most about the ring, 6 of 6 who used the TDF-only and TDF-FTC pod-IVR noted the fact that they could not feel it in their vaginas. Similarly, 5 of the 6 women who used the TDF-FTC-MVC pod-IVR noted they also liked that they could not feel it in their vaginas. Interestingly, when asked what they would change about the ring, 5 of 6 who used the TDF-only and TDF-FTC pod-IVR asked for a smaller, thinner ring, even though they could not feel it in their vaginas. When the women who used the TDF-FTC-MVC pod-IVR were asked what they would change about the ring, 4 of 6 noted that they thought the ring was fine the way it is, 1 noted she would make the ring smaller, and 1 noted she would like the ring to be a multi-purpose ring. This will be further explored in future studies.

All women (TDF-only IVR and TDF-FTC IVR) would use the pod-IVR if accessed by prescription (6 of 6) while 4 of 6 would use the pod-IVR if accessed over-the-counter. Three of the six women who used the TDF-FTC-MVC pod-IVR would use it if accessed by prescription and 4 of 6 would use the pod-IVR if accessed over-the-counter.

In the final survey, participants were asked, “If a ring could be designed to meet more than one need, which of the following uses would you want in one ring? In other words, what would you need a single ring to do for you? (Check all that apply).” Eight of ten women responded that they needed a ring that would protect them from one or more disease; two needed contraception alone. Additionally, nine of the ten participants responded that they would probably/definitely use the pod-IVR if it protected them as they needed it to. Only one remained unsure if she would use the pod-IVR in the future.

#### Confidence to use the pod-IVR

Overall, all six participants in Cohort 1 felt at least somewhat confident that they could insert the pod-IVR correctly, even with their first ring use. Other than one woman who felt that the second ring did not slide into her vagina as easily as the first ring, all participants either maintained their level of confidence or increased it. In Cohort 2, all participants were very or completely confident that they could insert the pod-IVR correctly, and the two women who used all three rings reported being very and completely confident that they could insert the IVR correctly.

The women were equally as confident in their ability to remove the pod-IVR, with 9 of 10 women being very or completely confident in being able to do so. The one woman who was only somewhat confident had only used one ring, so did not have the benefit of multiple opportunities.

All ten women were very or completely confident in their ability to access the pod-IVR, whether by prescription or over-the-counter. All participants were at least somewhat confident that they could keep the pod-IVR in for 28 days.

#### Important characteristics and/or functions of the pod-IVR

Women were asked what characteristics were important in an HIV prevention pod-IVR. Easy insertion was very or extremely important for all ten participants. Most of the women thought it would be very or extremely important that the IVR not be noticeable to themselves or their partners. Consistent with our previous data on sexual pleasure, the responses spread across the scale when asked whether it was important for the IVR to “make sex feel better.” The responses were also spread across the scale when asked if it was important that the ring was able to be removed during sex, or that they were able to remove the ring daily or weekly to rinse it off.

All participants felt that having no side effects was very or extremely important. And at the end of their participation, all participants thought it very or extremely important that the ring protect against other sexually transmitted infections.

### Qualitative results

#### First impressions of the pod-IVR

During the in-depth interviews (IDIs), participants were asked about their impressions on seeing the pod-IVR for the first time. All ten participants, regardless of cohort, felt the pod-IVR was bigger than they had anticipated, with three women discussing their initial concerns that the pod-IVR may not fit in their vagina. Interestingly, several noted, during their first experience inserting the pod-IVR, that it was more flexible than they expected it to be, referring to the experience of squeezing the IVR to insert it into their vagina.

#### Insertion process

Overall, all the women (10/10) successfully inserted the pod-IVRs into their vaginas following instruction from the study clinician. The insertion instructions provided verbally by the study clinician were detailed yet flexible, giving the women freedom to insert the product in a manner they felt most comfortable, whether trying various finger placements on the ring, or altering their body position for insertion. Thus, a variety of methods were used to insert the pod-IVRs. Four of the women found that laying down with knees bent allowed them to effectively insert the pod-IVR into their vaginas. Five of the women felt more comfortable in a standing position with one leg up on an elevated surface (e.g., clinic exam table, toilet seat) to insert the pod-IVR. One woman sat over the toilet and inserted the pod-IVR into her vagina in similar fashion to how she would insert a tampon. All the participants used a combination of their thumb, middle, and index fingers to squeeze the pod-IVR and insert it into their vagina.

Ease of Insertion and Confidence. Of the six women who used two pod-IVRs (cohort 1), four felt that their second experience inserting a pod-IVR was easier. For the other two women, their second experience inserting a pod-IVR proved more difficult than their first insertion experience. One participant had a harder time squeezing the second pod-IVR between her fingers, and noted that it did not seem as pliable as the first pod-IVR she had used. Another woman noted that the pod-IVR did not slide into her vagina as easily as the first ring had. Despite these differences, both felt confident they had inserted the ring correctly, and, once the clinician confirmed the placement of the pod-IVR for each of them, they reported that their confidence was firmly established. The two participants who used all three pod-IVRs noted that the experience of inserting the third pod-IVR was even easier than inserting the first two because they were familiar with the process and confident in their ability to correctly insert the ring.

#### Daily use experience: Pod-IVR awareness

Regardless of which pod-IVR was being used, all 10 participants, noted that they could not physically feel the pod-IVR in their vaginas during daily use. Common reflections included one woman describing her week as “uneventful” and another noting that it was almost as if she had not inserted the ring at all.

No participant mentioned that they could physically feel the ring once it was comfortably positioned. Three of 10 women said they were psychologically aware of their first pod-IVR. One of those three used all three pod-IVRs and reported being more comfortable with the use of each subsequent pod-IVR. She reported being very psychologically aware of the first pod-IVR, but feeling as though she had forgotten about it while using her third pod-IVR. She said she had to remind herself that the third pod-IVR was still inside of her vagina.

Seven of 10 were concerned that the ring may have fallen out since they were not able to feel it. Two of these women inserted a finger into their vagina to check that the ring was still in place and had not fallen out. One participant checked daily during her first ring use, but felt confident enough during use of the second ring that she only checked once midweek to make sure it was in place. Another participant checked for the ring with her finger on the first night of ring use. That experience allowed her to feel confident that the ring would stay in place and reported not feeling the need to check again. She added that she was not aware of the ring moving around or dropping in her vaginal canal as the week progressed. Although these participants had concerns that the pod-IVR may fall out, all the participants noted, during the IDI, being pleased that they could not feel it during daily use. One woman, after using her second of three pod-IVRs, said it was comforting to not feel the ring and to be able to go about her week as she normally would.

#### Daily use experience: Discharge

During in-depth interviews, all participants were asked about their experience of discharge while using the pod-IVR. Four of 10 participants experienced a day or more of discharge that they perceived as more than their usual discharge pattern during a given 7-day use period. The amount of discharge reported by these women ranged from “a few drops” to “a lot.” One of these women reported discharge/mucus toward the end of her first 7-day use period: she described the amount as “enough… to notice,” and added that there was no odor or color. Three women experienced discharge when wiping after using the toilet, while another noticed discharge on the ring following removal. Another woman who did not report greater than usual discharge patterns, also noticed discharge on the ring when she removed it. One other participant noted a small amount of discharge on her underwear during most of the 7-day use period. While she was the only participant who felt the need for a daily panty liner for the discharge, she did not experiencer this discharge pattern as more than her usual discharge. None of the participants initiated discussion of being bothered (or not) by the discharge.

#### Daily use experience: Hygiene

Change in Typical Hygiene Practices. Most of the study participants, 8 of 10, said pod-IVR use did not affect typical hygiene practices. The remaining two participants reported doing more to clean up while using the pod-IVR than typical for them because of the discharge they experienced. They felt the need to wipe their labia more and/or use sanitary pads.

#### Expulsion concerns: Toileting

Four of 10 participants were concerned that the ring would come out while having a bowel movement, either while bearing down, or that the bowel movement would cause the ring to shift or be dislodged. One of the women said she did not think about the ring except when she was going to the bathroom. She noted that the ring never felt like it was coming out, only that it concerned her. Another participant said she was initially concerned that the pod-IVR could come out if she was constipated and in a squatting position for too long, but noted that she stopped worrying about it after the first day of use. None of the women reported the ring ever coming out while using the bathroom; and each only had these concerns while using their first pod-IVR.

#### Expulsion concerns: Physical activity

Except for one woman who said she did not do any physical activity while using the pod-IVR, all other participants engaged in physical activities as usual including kick boxing, walking, running, and weight training. Although there were some concerns about the ring coming out while exercising, this did not happen for any of the participants, regardless of which ring was being used. One woman reported having concerns that the ring would fall out while kick boxing. She reported being quite aware of that possibility with her first pod-IVR, but noted that the ring did not come out. She addressed this concern for herself by taking the replacement ring with her to the gym during her second ring use period, just in case, but again reported that she did not feel the ring, nor did it ever come out during physical activity.

One of the women who used all three pod-IVRs experienced cramps while running when she was using her first pod-IVR; she ultimately limited the intensity of her physical activity when using the second and third pod-IVR to reduce the chance of getting cramps. She did not experience cramping with the second or third study rings. Another woman experienced cramping while lifting heavy weights at the gym on her third day of IVR use and decided to remove the IVR (she inserted the replacement IVR after working out).

#### Pod-IVR removal: Initial removal concerns

Participants had the opportunity to remove one or more rings, depending on their enrollment(s) in Cohorts 1, 2, or both. Prior to using their first and/or only ring in the study, half the women across cohorts had initial concerns about what the process of removing the ring from their vaginas would be like for them. Four women in Cohort 1 noted being nervous about the removal process with their first ring and unsure if they would be able to take the ring out of their vaginas. Three of these women noted that this was in part due to feeling uncertain as to how far the rings were inside their vaginas, which led them to think it could be challenging to grab onto the ring to remove it. This same sentiment was echoed by one of the women from Cohort 2.

#### Pod-IVR removal: Removal experiences

Women in both cohorts removed their pod-IVRs in a variety of positions, such as squatting (n = 8), sitting (n = 1), and lying down with legs in the air (n = 1). In describing their choice of position for removal of their first pod-IVR, two women shared that they had to change from the initial position they tried because they had a challenging time reaching for and feeling the ring in their vagina. One of these women initially tried standing up to remove the ring, but was not able to reach the ring with her fingers: she then sat on the toilet, which she said made it easier to reach for and feel the ring inside her vagina. The other woman initially tried to remove the ring in the same position as she inserted it, half-squatting with her feet wide apart; however, she couldn’t locate the ring inside her vagina with the resulting reach. Her second attempt had her lying down with her legs up; this allowed her to reach in far enough to feel the ring. All women in Cohort 1 removed their second pod-IVR using the same position that they removed their first ring. Further, the two women who used a third ring used the same position for removal as they did for their first and second rings: squatting and lying down.

One participant had to remove and replace her first ring on Day 3 of use and did so by sitting on the toilet and using her middle finger to hook the ring and pull it out. When she removed the ring again at the end of the 7-day use period, she decided to stand instead because she had more space to move her body around. She described this experience as being easier than the first time. One woman who used all three rings used her nondominant hand to remove the third ring: She reported being as confident using her nondominant hand, noting removal was just as easy.

In Cohort 2 (N = 6), one woman removed the ring as instructed by the clinician, which was standing, knees bent, and leaning over. She noted removing the ring went smoothly, taking 30 to 40 seconds in total. This woman compares the removal experience to finding string to remove a tampon that is sitting in the vagina higher than normal. In describing her experience, she said the ring “just slid without any discomfort, and there was no change in how [her] vagina felt. There was no pressure.”

Once women were able to find a comfortable enough position, a variety of combinations of fingers were used to hook the ring and pull it out, from using an index finger or middle finger to using a combination of fingers, such as the index and middle finger or index finger and thumb. One woman noted that she found it helpful to use non-latex gloves for cleanliness, but that she would also remove the ring without them if necessary. Two women said they found it helpful to push their forefinger inside their vagina both with gentle force and depth to reach the ring. Another woman described that it took a couple minutes of feeling around in the vagina for the ring.

#### Pod-IVR removal: Ease and confidence of removal

During the IDI, the women described when or what allowed their initial concerns regarding their ability to remove the pod-IVR to dissipate. For some, it was being able to physically feel the ring with their fingers, while for another it was finding a comfortable enough position. One woman thought that removing the ring was going to be painful because she had decided that the ring would have molded to her body and she was not going to be able to pull it out: she was pleasantly surprised to find it “just kind a [sitting] there.” Further, one woman said she was excited that she was able to remove the ring so easily and felt reassured that she would be able to pull it out again in the future. Another woman who only had a single opportunity to remove the pod-IVR noted that removing the ring was much easier than she expected.

When it was time for the women to remove the second ring, three participants said it was easy to remove the ring the second time. Two of those women, who had used all three pod-IVRs, said that their third ring removal was easy because of an increased familiarity with the process.

One woman thought it might be difficult for someone who had little or no experience inserting and removing things from their vagina. She suggested that longer nails would make it easier to dislodge the ring from behind the pubic bone. Another participant’s advice for ring removal: “calm yourself down, don't rush, take your time. It's not going to hurt to try to retrieve it, but it is best to go at a slower pace.”

#### Pod-IVR removal: Pod-IVR appearance when removed

Some women noted observations regarding the ring upon removal, from bodily secretions, to smell, to noticing (or not) the drug pod(s) in the ring. Five out of 10 women did not notice any change in the appearance or color of the rings themselves after use. Two of these women noted that the ring was still flexible and the same color as it was before they inserted it. Another of those women noted that, other than expected vaginal secretions on the ring, the pod-IVR was not different from what it looked like before insertion and that the color was still the same.

Eight out of the 10 women noted that the ring had a lot of mucus or vaginal secretions on it upon removal. Four women had a negative reaction to the vaginal fluid/discharge that was on the ring when they removed it, despite their having expected it. During the IDI, three women reported that they assessed for any smell to the ring when they removed it: one woman mentioned that she did not like the smell/odor of the used ring because it reminded her of medication, while the other two said that the rings did not have a smell/odor. Of the 5 women who mentioned the drug pods during their IDI, three women explained that they did not notice the drug pods on the rings when they took them out of their vaginas, while two women mentioned that they were able to see the drug pods.

One woman noted that she would want the ring to look different after removal than it did upon insertion, because she would feel confident that the ring was releasing drug if there were a visual cue for drug release. The other woman who used all three rings noted, in her final IDI, noted that there was a rusty color to the drug pods when the ring was removed. She thought this could have been from some bleeding she had (following a biopsy) and thought it was interesting that blood got into the pods. It is not clear if the participant noticed blood in the delivery channels, pods, or both.

#### Anticipations of pod-IVR use during menstruation: Willingness-to-use impacted by tampon use

When asked if they would be willing to use the pod-IVR during their menstrual period, 8 of 10 women said they would try it and 2 of 10 said they would prefer to take the pod-IVR out. Four of 10 women said they would only keep the pod-IVR inside their vagina during their menstrual period if they could also use a tampon. One of these four presumed that the tampon would not go in as far as the ring and so she did not foresee the tampon interfering with ring use: that said, she also had some concern that it would be uncomfortable.

One woman noted that she would prefer to take the ring out during menses to give her body a break from ring use. When asked about her thoughts on using the ring during her period, another woman noted that, while she would not be bothered by it because her menstrual flow is light, she could imagine it would not be as comfortable for women with heavy menstrual bleeding.

Three of the 8 women willing to use the pod IVR while menstruating were concerned about the ring coming out while removing the tampon. One women said she was worried that the tampon would get in the way of the ring inside of her vagina or that she would pull the ring out while removing the tampon.

#### Anticipations of pod-IVR use during menstruation: Effect of pod-IVR use on menstrual symptoms

While it remains an empirical question, three of the 10 participants had concerns that the drug(s) in the ring could make menstrual symptoms worse, such as more cramping, moodiness, and heavier periods. One also mentioned that she would be concerned if the ring affected the regularity of her periods, such as delaying her period or causing it to occur sooner.

#### Anticipations of pod-IVR use during menstruation: Effect of pod-IVR use on hygiene practices during menstruation

Three women reported having concerns about messiness caused by using the IVR while menstruating. One added that using the ring for a longer period of time would cause her to want to clean herself more often. Another participant who participated in both cohorts also said that she thought using the ring during menstruation would be messy and require more clean-up because, she presumed, menstrual blood would get into the pods. Two participants noted they would continue to use the ring during their menses if necessary, but they would use gloves to insert and remove the ring.

#### Anticipations of pod-IVR use during menstruation: Perceived product efficacy during menstruation

Four of 10 women had concerns with the effectiveness of the ring during menses. They were all concerned about the potency of the drug(s) and how menstrual blood flow could affect that. One was concerned that some of the drug would leave her vagina with her menstrual fluids. Another raised concern that the tampon would absorb some of the drug(s) and consequently decrease the effectiveness of the ring.

One women did not have a concern with having the ring in her vagina during menses because she surmised that the moisture during her period would help the drug(s) in the ring come out more [and get distributed to the tissue]. Importantly, she simultaneously had concerns about the efficacy of the ring overall, because she believed that it would be counterproductive for menstrual blood that contains the drug(s) to come out of the body.

#### Anticipations of pod-IVR use during menstruation: Length of pod-IVR use and menstrual period

When asked about their thoughts on using a pod-IVR for longer periods of time, such as 28, 60, or 90 days, one woman said she would not need to take the ring out during her period to wash it: She would just forget about it. Another woman said that, for women wanting to have a regular period, she could see them wanting to remove the ring once a month for their period but thought they would be able to use a 28-day pod-IVR. For others on contraception that suppresses their period, she said they may want to use a 90-day pod-IVR. One women added that if the ring was a 60- or 90- day ring, it would be nice for those with a heavy period to be able to remove the ring and clean it at the end of the menstrual cycle. Another said she would use the pod-IVR for 28 days and would take it out for her period. She added that she would only use the ring for 60 or 90 days if she could use a tampon during menses: otherwise, she would not use the ring because she does not like using sanitary napkins.

#### Anticipations of sexual experience and pleasure: Awareness of pod-IVR during sex

The current study involved sexually abstinent participants. However, during the IDIs, participants were asked to anticipate how the pod-IVR might be experienced during sex. Most felt it was difficult for them to speculate on what the pod-IVR would feel like during sex without having had the actual experience. That said, a range of opinions emerged on whether (or not) the IVR would be felt during vaginal sex by either partner. After using the first pod-IVR, all six of the participants in Cohort 1 thought that they would be able to feel the ring during sex and that their sexual partner would feel it also. They were concerned the ring would cause discomfort, or that it might come out of the vagina during sex. After using the second pod-IVR, only two of these same six women thought they would feel the ring during sex. The four women who changed their minds attributed the change to not physically feeling the pod-IVR at all during their week of use. All six women in Cohort 2 (including the two women who participated in Cohort 1) expressed similar concerns. One of the new participants noted that she anticipated that both she and her sexual partner would feel the IVR during sex, but that it wouldn’t necessarily be an inconvenience.

#### Anticipations of sexual experience and pleasure: Removing the pod-IVR prior to sex

A few participants liked the idea of being able to take the pod-IVR out during sex if it became bothersome, but wondered if it might become annoying to take the ring out every time they had sex. Another participant expressed concern with removing the IVR prior to sex: she felt that it might not be hygienic to remove and reinsert the ring.

#### Anticipations of sexual experience and pleasure: Pleasure during sex

The participants also had a range of opinions on whether (or not) the pod-IVR would increase or decrease sexual pleasure for either partner. Two participants noted that potential pressure caused by feeling the ring in the vagina during sex could heighten sensation and pleasure for a woman. Two other participants agreed that the ring could potentially increase sexual pleasure for either partner, but were unsure how exactly. These women felt positively about the pod-IVR increasing sexual pleasure. Another participant noted that she would not want to use the IVR if it caused sex to be less pleasurable.

#### Anticipations of sexual experience and pleasure: Covert use

The participants mentioned several reasons why they could and could not use the pod-IVR covertly or discreetly. Five out of 10 women stated that they could not use the ring covertly because they believed their partner would be able to feel the ring during sex. One of these women mentioned that she would have to tell her partner; otherwise, he would be upset if he found out about her using the ring merely by feeling it during sex. Another of the five women said that since it was so easy to find the ring to remove it, she thought that her partner would be able to notice the ring during sex just as easily. However, this same woman also said that if someone did not tell their partner about the ring and he felt it during sex, it would be easy to say that the pod-IVR is a birth control ring. Personally, this woman would want to tell her partner beforehand, especially if she used the ring regularly.

Four of these same women, and one additional woman gave reasons as to why the pod-IVR could be used covertly. Two said that, as long as you can insert and remove the pod-IVR privately, the ring could be used without others knowing. Another said that the ring is firm but not rigid and it would be unnoticeable during sex; she did not think she would feel the ring. In addition, she said that since men have sex with women who have IUDs and they often cannot feel the string during sex, she believes she could use the pod-IVR if she did not want her partner to know.

While these women gave insight into why a ring could and could not be used covertly, several women also provided insight into why the ring would, or would not be used covertly, regardless of the potential for covert use. Eight women noted that they personally would not want to use a ring covertly. Three of these noted that it would be more comfortable for them if a partner knew about the ring, especially if they were using the ring regularly. One said that, because of her own personal views on relationships and sexual partners, she would want to tell her partner about her ring use. Another mentioned that covert ring use with a consistent partner could indicate a lack of trust. Of the two women who anticipated being able to use the pod-IVR covertly, one said she would not want to tell her partner but might if he felt the pod-IVR during sex. The other woman said she would not feel a need to tell her partner that she was using the ring unless he could feel it during sex and commented about it. If he commented on the ring, she felt like she would be able to tell him it was for medicinal purposes and “no big deal.”

To the contrary, seven of the women, including all those just mentioned, provided reasons why they might want a ring that could be used covertly. One woman said she was concerned a partner might not want to use a condom if she were using the ring; this discussion led her to think that she would not tell a new partner about her ring use so that he would still be willing to use a condom. Another woman talked about how she liked the idea of being able to use prevention medication without needing to tell anyone or being able to pass it off as contraception or a sex toy.

#### Portability and discreetness

Six women said it would be easy to store the ring discreetly and travel with it. According to one, it would be easy to put the ring in a purse, backpack or jacket pocket, but not a pants pocket because the ring is too thick and people might see the circular shape and think she had a condom in her pocket. In addition, one woman mentioned that she liked the clear bag that it came in, and described it as portable.

One woman was concerned that the ring could not be stored discreetly because the trial packaging being so big. Five out of 10 women said that if the ring was made smaller or the packaging was smaller, similar to that of panty liner packaging or a make-up compact, it could be more discreet.

## Discussion

The user experience is an important consideration in uptake and continued use across myriad health-related products.[21] This is true for HIV prevention [7, 9, 22–24] technologies, as well as current efforts to advance multipurpose prevention technologies (MPTs) for sexual health.[8, 25] The results here, documenting the first data regarding user experiences with of a novel drug delivery platform, the pod-IVR are particularly important. As development of intravaginal systems for delivery of preventive agents continues, the importance of understanding the user experience as part of the preclinical design process is being acknowledged.[26, 27] Our results provide evidence to support continued development of this platform, and offer a considered look at issues that will likely impact the development of vaginal products for long-term use in prevention of HIV, as well as new contraceptive methods and MPTs.

Overall, participants were highly satisfied with the pod-IVR and its potential. Once inserted and positioned correctly, participants could not physically feel the pod-IVR during daily use. Insertion and removal confidence was relatively high and, for most, increased with experience. When each participant was asked to consider the pod-IVR as one which could meet their specific sexual and reproductive health needs (HIV prevention, STI prevention, contraception or some combination) all but one participant reported being willing to use it in the future.

Assessing the ideal length of sustained residence and drug-release for HIV and MPTs has been the subject of inquiry for some time, prompting development of pericoital, daily use, and long-term or sustained delivery products. Specifically with respect to IVRs, development continues with 30-, 60-, and 90-day targets, as well as products that would delivery protection for a year or more.[4, 6, 9, 28] Participants in this 7-day study, indicated that their willingness to use the ring would decrease as longer use periods were considered. These data are compelling, given the active assumptions in the current prevention product development pipeline. Unlike the women in this study, otherwise healthy post-menopausal women using vaginal rings to alleviate vaginal dryness or urinary discomfort have tended to find a long-term IVR acceptable and prefer them to some other drug delivery systems.[29–30] What is it about long-term use that reduces motivations for use in HIV prevention? It remains unclear whether psychosocial motivations active in IVR-based prevention product use might differ from those of IVR-based treatment-associated use, or if motivations are drug delivery system-dependent. This is an important caution, indicating the importance for assessment of actual long-term ring use.

Whether women will want to continually use an intravaginal prevention product for several months remains an empirical question. In-depth inquiries, like those reported here, are essential to answering that question. These data provide more nuanced detail of relevant user experiences. Some support overall willingness-to-use, while others suggest potential challenges to ring use, potential opportunities to optimize future clinical trial procedures, and opportunities to enhance real world use by addressing participant/patient education, the learning process associated with ring insertion and removal, and confidence to use. The narrative data provide insight into salient user experiences and the meanings they derive from those experiences.

Typical side effects associated with systemic (i.e., oral) dosing of antiretroviral agents are likely to be avoided with IVRs due to the low systemic drug exposure. This, in itself, is an advantage of IVRs, but other potential side effects (cramping, discharge) noted in this study may impact user behavior and, hence, product effectiveness. For instance, cramping likely associated with poor ring positioning led a participant to avoid her normal high intensity running routine in order to avoid additional cramping. While this is tolerable for a participant in a 7-day study, it is unlikely, and undesirable, that a user would allow continued ring use to change her normal physical activity in longer (e.g., 30-day) use studies or real-world use. These data also point to the importance of correct ring placement, and effective communication and education about placement, given its importance for a long-term resident ring.

As studies proceed, it will be important to ascertain the amount of time a user could safely remove the ring (before re-inserting the same ring or inserting a new ring) and maintain preventive effect. Might it be possible for the ring to be removed for exercise, or sexual intercourse, if desired? If so, instructions for checking proper placement, repositioning the ring, or removing and replacing the ring could be developed that would both optimize drug effect and allow for greater flexibility in the use regimen, thus optimizing overall adherence to ring use in clinical trials and beyond.

In clinical trial settings such as this, especially those with an in-depth interview element, participants naturally can become keen observers of their bodies. For instance, in this study, women reported experiences with vaginal discharge. Some described this as more than their typical discharge, while others did not. What they notice is not necessarily out of their normal experience, but they ascribe the experience to the study product instead of to their normal vaginal hygiene processes. The vigilance elicited by study participation is highly advantageous for helping researchers identify product-related red-flags and exploring the nuance of user experiences to support product adherence, but it presents a challenge if we, too, assume their experience is study product related rather actually gathering data confirm or deny that association.

It is also important to note that, whether vigilant observations or normative reports, users will make meaning of the experiences they have while using a study product. And whether the meaning they derive is clinically accurate or not, that meaning-making will impact their use behavior.[7, 31, 32] Ultimately, such observations should facilitate the next generation of clinician-participant communication materials and/or procedures for later clinical trials or in subsequent patient education materials.

Clinician instructions were important to initial learning and creating the opportunity for an effective learning process. Note should be taken of the range of body positions, hand/finger maneuvers, and changes in those positions and maneuvers over time as participants learned to inert and remove the ring. Without exception participants valued the instructions provided by the study clinician in this regard. Key within those instructions was the permission given to the participant to explore and be flexible in their own best practices. Participants felt encouraged and supported, even when they harbored concerns about their abilities to insert or remove the ring correctly. This instruction is important to adherence and confidence in use, empowering participants to try new positions with their second or third ring insertion.

### Study strengths and limitations

These data were captured within a pre-phase 1 first-in-human clinical study context. Such a context demands a small sample size for each pod-IVR evaluated. While this is not an inherent limitation for qualitative inquiry, especially in cases where a single participant completes more than one ring evaluation and can provide comparative data, the quantitative data cannot be statistically analyzed due to a lack of statistical power. That said, by combining quantitative and qualitative data, and considering quantitative data on an individual as well as group basis, this mixed methods user experience study provides valuable insights into the potential opportunities and challenges afforded by this drug delivery platform. The safety/PK trial context for these data may have impacted user experiences. Participants underwent vaginal biopsies and other clinical evaluations, and involvement in a clinical trial itself could have altered participants’ experience of the pod-IVR, whether negatively or positively.

As can be seen in Table 1, all 10 women could reflect on a single 7-day experience during the in-depth interview; 6 women could reflect on their pod-IVR experiences in the course of two 7-day use periods and draw comparisons between those two experiences, and 2 women who evaluated all three pod-IVRs could further reflect on three 7-day use periods. Each scenario is important for different reasons. In the case of all 10 women and their first in-depth interview, we can most closely approximate the range of experiences and outcomes relevant to “trying” a product. For the 4 women who completed a second pod-IVR use period, and the two who completed a third, we have the opportunity for both greater learning and familiarity to play a role in users’ perceptions and opinions, and for user comparisons between products where the drug delivery platform remained the same but pharmaceuticals changed.

While a cohort of generally healthy women who report little to no risk for HIV infection have a different risk context in which to evaluate the product with respect to willingness-to-use in a general sense, the range of user experiences studied here are largely relevant to any women who would be using these products. That is, that they felt or experienced the pod-IVR in certain ways likely is relevant across risk contexts: what is potentially differently relevant across risk contexts is what meaning users make of those experiences. This pre-phase 1 study, importantly, identifies the range of insertion, removal and daily use experiences of pod-IVR users, which can now be explored in detail within other user cohorts.

### Implications

Moving forward, issues raised in this 7-day pod-IVR use study will be elucidated in greater depth over longer use periods (e.g., 28-day use period). What women want in a prevention product (HIV and/or STI prevention, and/or contraception) will be further explored, as will the length of use most preferred as a function of product indication. We will continue to explore potential best practices for both insertion and removal instructions. The impact of ring use on physical activity, and hygiene behaviors, as well as ring experiences during menses and sex will also be explored in detail. Within the domain of ring use during menses, it will be important to specifically consider feminine hygiene practices such as tampon use and (1) how tampon use might impact ring effectiveness, and (2) what demands ring use parameters place on hygiene practices. This will likely include the development of use instructions specific to tampon users. Given the import of partner involvement and experiences in sex, we also plan to explore the impact of ring use on the sexual partner’s experience. This, of course, should include specific sensory use experiences of condom-protected and condomless sex, as well as circumcised and uncircumcised male partners.
